# Urothelium muscarinic activation phosphorylates CBS^Ser227^ via cGMP/PKG pathway causing human bladder relaxation through H_2_S production

**DOI:** 10.1038/srep31491

**Published:** 2016-08-11

**Authors:** Roberta d’Emmanuele di Villa Bianca, Emma Mitidieri, Ferdinando Fusco, Annapina Russo, Valentina Pagliara, Teresa Tramontano, Erminia Donnarumma, Vincenzo Mirone, Giuseppe Cirino, Giulia Russo, Raffaella Sorrentino

**Affiliations:** 1 Department of Pharmacy, University of Naples, Federico II, Via D. Montesano, 49, Naples, 80131, Italy; 2 Interdepartmental Centre for Sexual Medicine, University of Naples, Federico II, Via Sergio Pansini 5, Naples, 80131, Italy; 3 Department of Neurosciences, Human Reproduction and Odontostomatology, University of Naples, Federico II, Naples, 80131, Italy

## Abstract

The urothelium modulates detrusor activity through releasing factors whose nature has not been clearly defined. Here we have investigated the involvement of H_2_S as possible mediator released downstream following muscarinic (M) activation, by using human bladder and urothelial T24 cell line. Carbachol stimulation enhances H_2_S production and in turn cGMP in human urothelium or in T24 cells. This effect is reversed by cysthationine-β-synthase (CBS) inhibition. The blockade of M1 and M3 receptors reverses the increase in H_2_S production in human urothelium. In T24 cells, the blockade of M1 receptor significantly reduces carbachol-induced H_2_S production. In the functional studies, the urothelium removal from human bladder strips leads to an increase in carbachol-induced contraction that is mimicked by CBS inhibition. Instead, the CSE blockade does not significantly affect carbachol-induced contraction. The increase in H_2_S production and in turn of cGMP is driven by CBS-cGMP/PKG-dependent phosphorylation at Ser^227^ following carbachol stimulation. The finding of the presence of this crosstalk between the cGMP/PKG and H_2_S pathway downstream to the M1/M3 receptor in the human urothelium further implies a key role for H_2_S in bladder physiopathology. Thus, the modulation of the H_2_S pathway can represent a feasible therapeutic target to develop drugs for bladder disorders.

The urinary bladder has two important functions, the storage of urine and emptying. Storage of urine occurs at low pressure, which implies that the bladder relaxes during the filling phase as opposite to the emptying phase. The wall of the bladder, is composed of three layers i) the outer layer, or detrusor muscle, consisting of a mass of interlacing smooth muscle fibers and elastic tissue, ii) the middle layer, or lamina propria, a thin layer of areolar tissue that connects the muscular with the mucous layer iii) the inner layer, or urothelium, composed of transitional epithelium. The urothelium in the past was considered as a passive mechanical barrier protecting the body from the toxic effects of urine, but in recent years it has been recognized as a functional structure. Actually, it is now clearly defined that the urothelium has sensory and secretory functions^1^,^2^,^3^,^4^,^5^,^6^. One of the most important accepted function of the urothelium is to modulate the detrusor activity. Indeed, it is now clear that urothelium exerts a significant inhibitory effect on detrusor contractility. What is still matter of debate is the nature of the mediator(s) secreted by the urothelium that operate this control. At the present stage it has been hypothesized the presence of an urothelium-derived relaxing factor(s), namely UDRF whose nature has not been as yet defined^7^,^8^,^9^,^10^. So far it has been suggested, from *in vitro* studies, that UDRF(s) is released within the urothelium upon muscarin (M) receptors stimulation but its chemical nature is still unknown^9^,^10^.

Recently, it has been proposed a new signal molecule in the control of bladder tone i.e. hydrogen sulfide (H_2_S)^11^. H_2_S, is the third gaseous transmitter along with nitric oxide (NO) and carbon monoxide, formed by L-cysteine mainly through the action of cysthationine-β-synthase (CBS) and cysthationine-γ-lyase (CSE)^12^,^13^,^14^,^15^. It is endogenously produced in urinary bladder of trout, mouse, and rat implying that the contribution of this pathway to bladder function is conserved in different species including human^16^. Moreover, animal pre-clinical studies showed that H_2_S causes smooth muscle relaxation and/or contraction depending on the species and experimental conditions used^17^,^18^,^19^,^20^. Of note, human functional studies have demonstrated that both sodium hydrogen sulfide (NaHS, an H_2_S donor) or L-cysteine (the H_2_S precursor) relax human bladder strips^11^. It has also been reported that H_2_S relaxing effect on human bladder involves potassium-ATP dependent channel activation^17^. Both enzymes CBS and CSE are constitutively expressed either in human urothelium or in detrusor muscle and are able to generate H_2_S^11^,^21^. More recently, a key role for CBS in human urothelium has been proposed^21^. Indeed, CBS rather than CSE is the main enzymatic source of H_2_S biosynthesis in human urothelium. The activity of CBS within the urothelium is regulated by phosphorylation. Particularly, the enzyme is shifted to an higher status of activity by phoshorylation at Ser^227^, in a cyclic guanosine monophosphate/protein kinase G (cGMP/PKG)-dependent manner^21^. In other words, an increase in cGMP promotes an enhancement in H_2_S generation that in turn relaxes human bladder. Taken together these findings, H_2_S appears to be a phylogenetically ancient and versatile regulatory molecule involved in the regulation of bladder homeostasis. For this reason, the H_2_S pathway could represent an attractive target for the development of new pharmacological tools. Nevertheless, more studies are needed in order to clearly define the role of H_2_S signaling in human bladder pathophysiology. Here, by using human urothelium and the urothelial cell line T24, we have investigated i) whether M receptor activation is downstream triggered the H_2_S pathway and ii) if H_2_S can be considered as an UDRF.

## Results

### Carbachol-induced contraction is affected by H_2_S in human bladder strips

**Figure 1:**
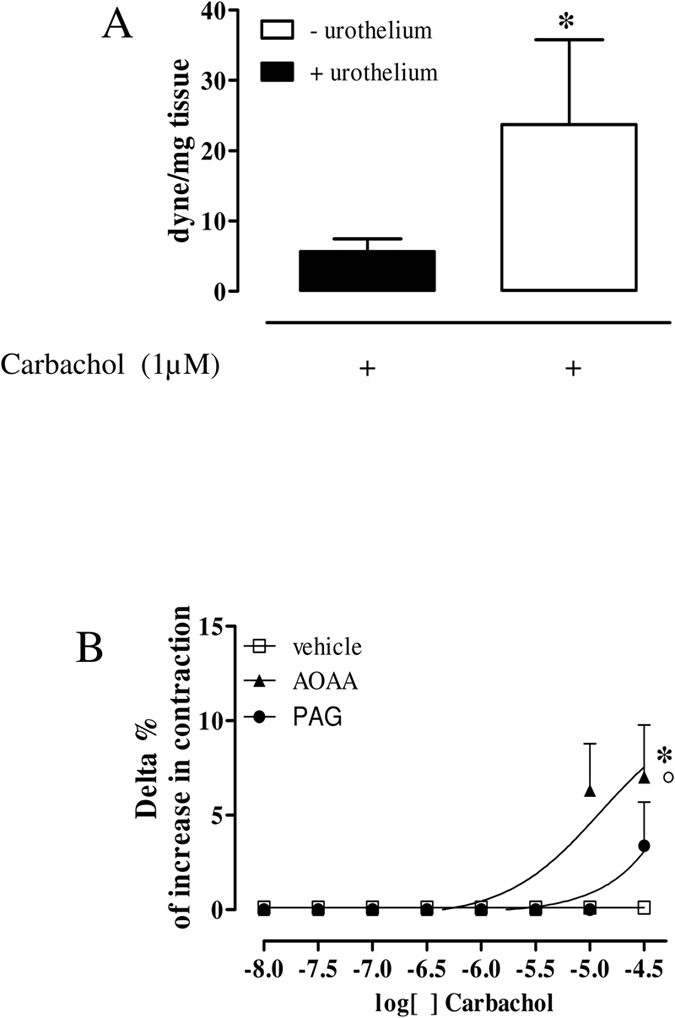
Carbachol induces contraction in human bladder strips. (**A**) Carbachol**-**induced contraction is significantly higher in human bladder strips without urothelium compared to strips with intact urothelium (*p < 0.05). Data are calculated as dyne/mg of tissue. (**B**) Delta % of increase in contraction induced by carbachol (10 nM–30 μM) in bladder strips with intact urothelium is not modified by PAG (10 mM), a CSE inhibitor. AOAA (1 mM), a CBS inhibitor, significantly increased carbachol induced contraction (*p < 0.05). AOAA effect was significantly higher than PAG (°p < 0.05). All data are expressed as mean ± SE of four different experiments.

The urothelium removal significantly potentiates carbachol-induced contraction (1 μM) (*p < 0.05; Fig. 1A). This finding confirms that the urothelium contributes to bladder homeostasis by producing relaxing factors. In order to assess the possible involvement of the endogenous H_2_S in carbachol-induced contraction, human bladder strips with urothelium were contracted with carbachol (10 nM–30 μM) in presence of aminooxyacetc acid (AOAA, 1 mM), a CBS inhibitor, DL-propargylglicine (PAG, 10 mM), a CSE inhibitor, or vehicle. AOAA but not PAG significantly increases carbachol-induced contraction (*p < 0.05; Fig. 1B). Of note, the effect of AOAA is higher compared with PAG treatment (°p < 0.05; Fig. 1B). The vehicle does not affect the concentration-dependent contraction induced by carbachol (Fig. 1B).

### Carbachol increases H_2_S production in human urothelium and T24 cells

**Figure 2:**
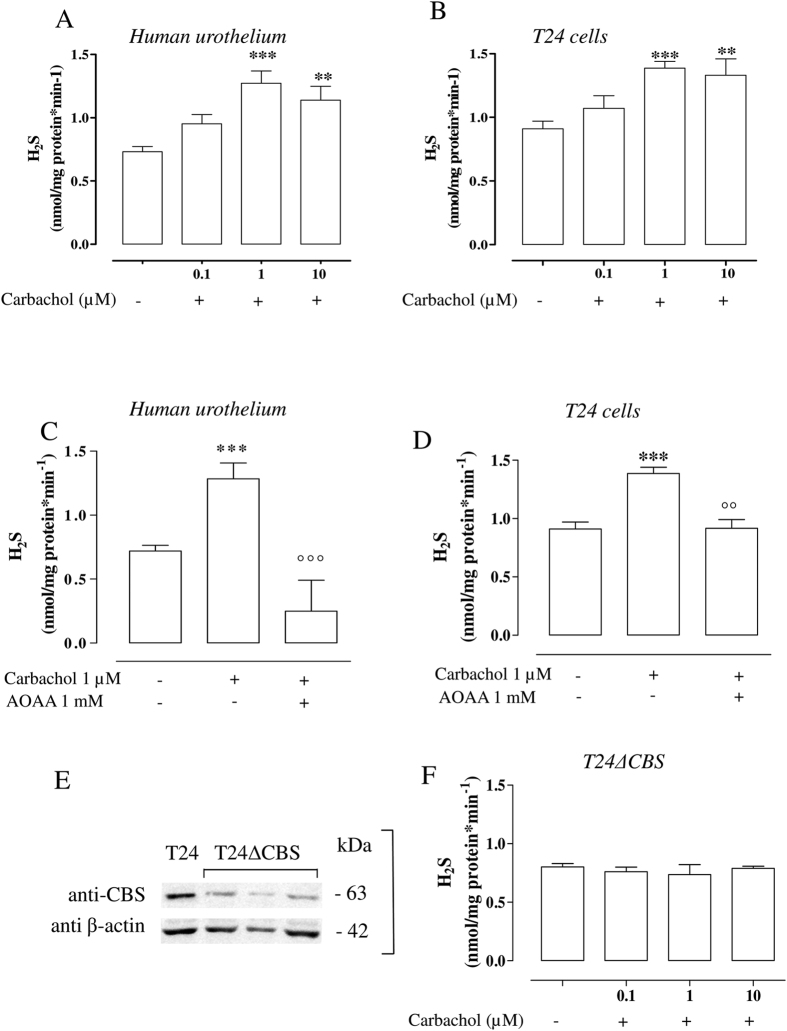
Carbachol increases H_2_S production in human urothelium and T24 cells. (**A**,**B**) Carbachol (0.1, 1 and 10 μM) causes a significant increase in H_2_S generation in human urothelium and in T24 cells, respectively (**p < 0.01 and ***p < 0.001 vs vehicle). (**C**,**D**) AOAA (1 mM), a CBS inhibitor, abolishes the increase in H_2_S production induced by carbachol (1 μM) in human urothelium (***p < 0.001 vs vehicle, °°°p < 0.001 vs carbachol) and in T24 cells (***p < 0.001 vs vehicle, °°p < 0.01 vs carbachol). (**E**) Western blot analysis for CBS in T24 cells or T24ΔCBS cells, using β-actin as a protein loading control. (**F**) H_2_S production in T24ΔCBS is not modified by carbachol (0.1, 1 and 10 μM) compared to vehicle. Data are calculated as nanomoles per milligram of protein per min and expressed as mean ± SE of four different experiments.

The incubation with carbachol (0.1, 1 and 10 μM) causes a significant increase in H_2_S generation compared with vehicle (**p < 0.01, ***p < 0.001) in both human urothelium (Fig. 2A) and T24 cells (Fig. 2B). To evaluate the involvement of CBS in H_2_S production induced by carbachol (1 μM), we have incubated human urothelium as well as T24 cells with AOAA (1 mM). CBS inhibition significantly reduces the increase in carbachol-induced H_2_S production in human urothelium (°°°p < 0.001 vs carbachol; Fig. 2C) as well as in T24 cells (°°p < 0.01 vs carbachol; Fig. 2D). In order to confirm the CBS role in human urothelium, as previously demonstrated^21^, we have stably silenced CBS in T24 cells namely T24ΔCBS (Fig. 2E). T24ΔCBS produces a detectable amount of H_2_S in basal condition (Fig. 2F). When T24ΔCBS cells are stimulated with carbachol (0.1, 1 and 10 μM) the increased H_2_S production is blunted (Fig. 2F) as compared to control cells (Fig. 2B).

### Carbachol phosphorylates CBS through cGMP/PKG pathway

**Figure 3:**
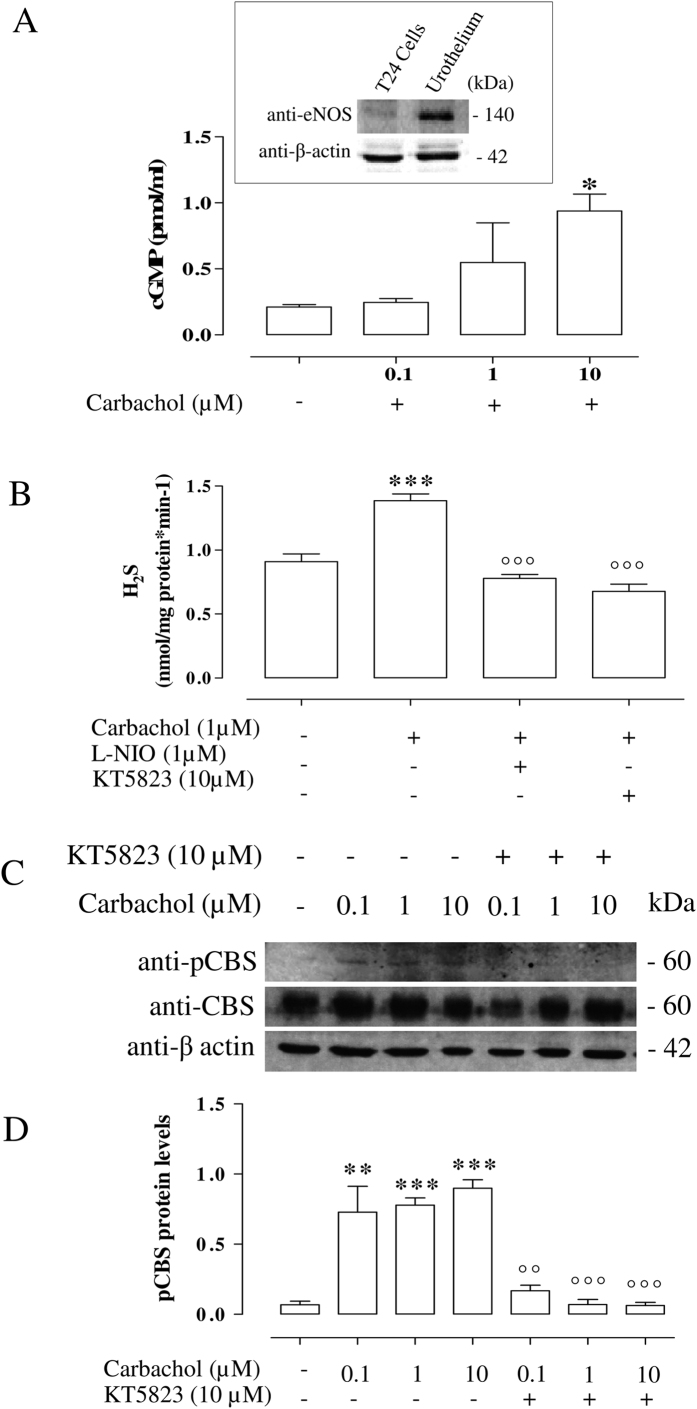
Carbachol-phosphorylates CBS involving NO/cGMP/PKG signaling in T24 cells. (**A**) Western blot for eNOS in T24 cells or human urothelium (upper). Carbachol stimulation (0.1, 1 and 10 μM) increases cGMP production compared with vehicle (*p < 0.05). (**B**) L-NIO (1 μM) or KT5823 (10 μM), eNOS or PKG inhibitors respectively, reduces carbachol-induced increase in H_2_S production (°°°p < 0.001 vs carbachol). (**C**) Western blot analysis for CBS, pCBS (ser^227^) in T24 cells stimulated with vehicle, carbachol (0.1, 1 and 10 μM) or in presence of KT5823 (10 μM), a PKG inhibitor. β-actin was used as a protein loading control. (**D**) Carbachol (0.1, 1 and 10 μM) significantly increases CBS phosphorylation compared with vehicle (**p < 0.01 and ***p < 0.001 vs vehicle). KT5823 (10 μM), a PKG inhibitor, inhibits CBS phosphorylation induced by carbachol (°°p < 0.01; °°°p < 0.001 vs carbachol). Western blot analysis data are quantified by densitometric evaluation and expressed as mean ± SE of four different experiments. H_2_S production data are calculated as nanomoles per milligram of protein per min and expressed as mean ± SE of four different experiments.

Firstly, we showed the presence of eNOS in T24 cells or in human urothelium (Insert in Fig 3A). Since human CBS is activated through cGMP/PKG-dependent phosphorylation at Ser^227,^^21^ we have evaluated whether carbachol increases cGMP in T24 cells. Carbachol stimulation (0.1, 1, 10 μM) significantly increases cGMP levels (*p < 0.05 vs vehicle; Fig 3A). As, cGMP/PKG is downstream from the NO signal, we used L-NIO (1 μM), as eNOS inhibitor, or KT5823 (10 μM), as PKG inhibitor. Both significantly inhibit the increase in H_2_S production induced by carbachol in T24 cells (°°°p < 0.001 vs carbachol; Fig 3B).

Finally, western blot analysis shows that carbachol triggers a significant increase in p-CBS^227^ compared to vehicle (**p < 0.01 and ***p < 0.001; Fig 3C,D). The involvement of cGMP/PKG pathway is confirmed by the finding that CBS phosphorylation is significantly reduced upon PKG inhibition (°°p < 0.01, °°°p < 0.001 vs carbachol; Fig 3C,D).

### M1 and M3 receptors are involved in carbachol-induced increase in H_2_S production in human urothelium and T24 cells

**Figure 4:**
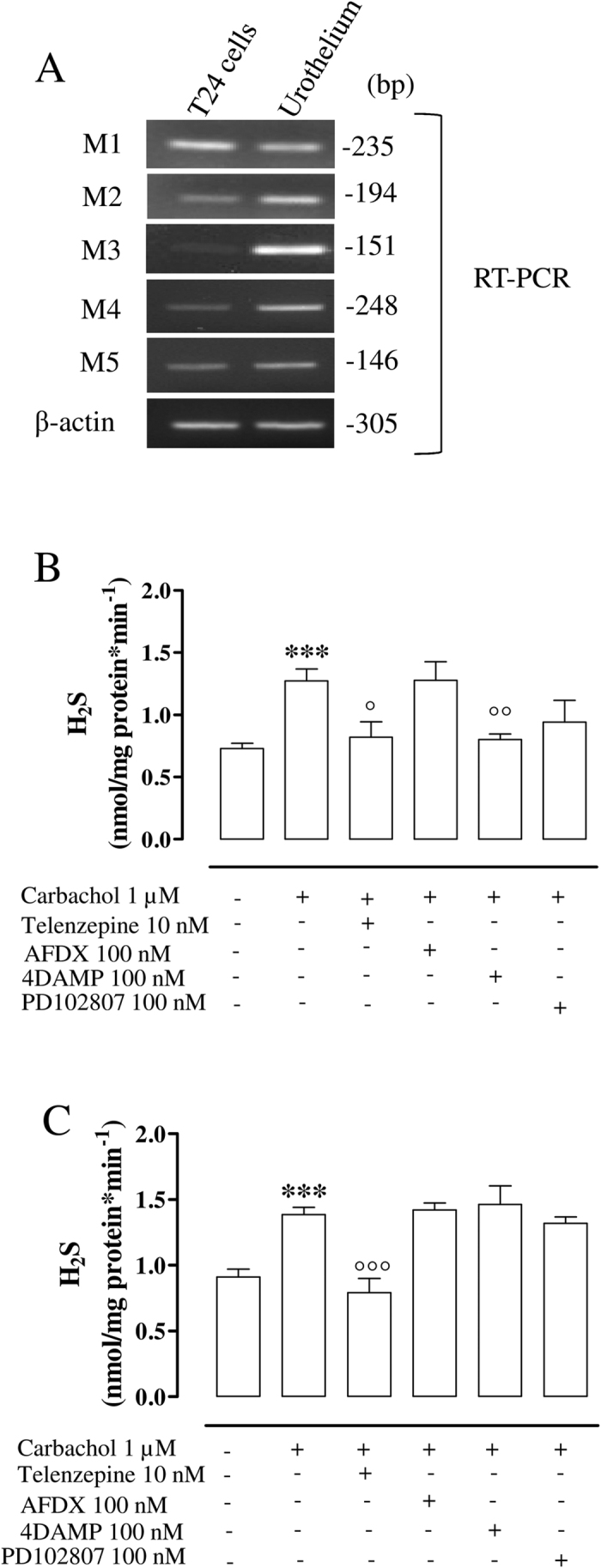
Muscarinic receptors involvement in carbachol induced increase in H_2_S production. (**A**) Semi quantitative RT-PCR for muscarinic receptors M1, M2, M3, M4, M5 in T24 cells and human urothelim respectively. (**B**) Telenzepine (10 nM) or 4DAMP (100 nM), M1 and M3 antagonists respectively, significantly reduces carbachol-induced increase in H_2_S production in human urothelium (***p < 0.001 vs vehicle, °p < 0.05 and °°p < 0.01 vs carbachol). (**C**) The blockage of M1 receptor (telenzepine 10 nM) markedly reduces H_2_S generation compared with carbachol in T24 cells (***p < 0.001 vs vehicle, °°°p < 0.001 vs carbachol). H_2_S production data are calculated as nanomoles per milligram of protein per min and expressed as mean ± SE of four different experiments.

M receptors (M1, M2, M3, M4 and M5) are expressed as mRNA either in human urothelium or in T24 cells (Fig. 4A). In order to address the involvement of a specific subtype of M receptor in H_2_S generation, we have used selective M antagonists. Notably, the blockade of M1 or M3 receptor, with telenzepine (10 nM) or 4-DAMP (100 nM) respectively, abrogates carbachol-induced increase in H_2_S production in human urothelium (°p < 0.05 and °°p < 0.01 vs carbachol; Fig. 4B). In T24 cells, only the blockade of M1 receptor with telenzepine, significantly reduces carbachol-induced increase in H_2_S production (°°°p < 0.001 vs carbachol; Fig. 4C).

## Discussion

The urinary bladder expresses all M receptor subtypes that are widely and heterogeneously distributed, in the different area of the bladder^22^. Activation of M receptors on the detrusor muscle triggers the contractile response necessary for urinary bladder voiding. However, M receptors are also involved in relaxing bladder^9^,^10^,^23^. This Janus effect (doubled edged effect) implies that different downstream signaling as well as mediators must be involved following M receptors activation within the bladder structure. Here, we have addressed the role of H_2_S as possible downstream mediator following activation of M receptors within the urothelium. Indeed, endogenous H_2_S is a signaling molecule which participates in the regulation of various physiological processes in human urogenital-tract^11^,^16^,^17^,^21^,^24^,^25^,^26^. The choice of the urothelium stems from the finding that CBS is markedly expressed in the urothelium as compared to CSE and that its activity is specifically enhanced upon phosphorylation^21^. Indeed, CBS is phosphorylated at Ser^227^ in a cGMP/PKG-dependent mechanism^21^. We have shown that the human bladder, as also reported by others, contracts to carbachol *in vitro* and this contractile effect is enhanced upon urothelium removal^9^,^10^,^27^,^28^. In addition, the inhibition of CBS, significantly increased carbachol-induced contraction in human bladder strips with intact urothelium while, PAG, a CSE inhibitor, did not significantly modify carbachol effect. This data indicates that i) urothelium dynamically contributes to pacing the contractile cholinergic stimulation and ii) endogenous production of H_2_S is involved in bladder tone. This hypothesis is further sustained by the finding that carbachol causes a significant concentration-dependent increase in H_2_S production in human urothelium. As it happened in the functional study, the inhibition of CBS blocks carbachol-induced H_2_S production in human tissue. Therefore, H_2_S is involved as downstream signaling mediator following cholinergic stimulation in urothelium. In order to further address this issue, we used the T24 cell line that mimics the human urothelium biochemical profile for what concerns the H_2_S pathway, as previously demonstrated^21^. T24 cells express all M receptor subtypes and, as expected, carbachol causes a significant enhancement in H_2_S production in T24 cells that is reversed by AOAA. Since AOAA is not selective for CBS^29^, in order to confirm the role hypothesized for CBS-derived H_2_S, we silenced CBS in T24 cells. In the T24 CBS silenced cells challenge with carbachol failed to increase H_2_S production confirming a key role for CBS-derived H_2_S. Thus, following activation of the M receptors the downstream activation of CBS is necessary in order to trigger H_2_S production. Since PKA is almost absent in human urothelium as well as in T24 cells we have investigated the possible involvement of cGMP^21^. A role for the NO/cGMP in relaxing effect specially in bladder neck and urethra has been demonstrated^30^. Here, the activation of M receptors by carbachol causes an increase in cGMP levels in T24 cells. In parallel, the western blot analysis confirmed the presence of eNOS in the urothelium. Thus, our results suggest a role for NO/cGMP in detrusor muscle dome through urothelium M stimulation. In order to evaluate the involvement of the NO/cGMP/PKG pathway following M receptor activation in urothelium we modulated eNOS or PKG activity by using the selective inhibitors, L-NIO or KT5823 respectively. Both treatments inhibited carbachol-induced H_2_S production bringing back to control levels. As expected, the increased cGMP production translated into an increased phosphorylation of CBS at Ser^227^ in T24 cells. Therefore, taken together all these findings imply that following M receptor stimulation eNOS/cGMP pathway is activated and cGMP produced in turn phosphorylates CBS in a PKG-dependent manner, thereby increasing H_2_S production. In order to investigate which M receptor (M1–M5) was involved in carbachol-induced H_2_S generation, we used different selective M1 to M4 receptor inhibitors since there are no available selective M5 receptor inhibitors. In T24 cells only telenzepine, an M1 antagonist, reduced H_2_S production while in human urothelium, the blockade of either M1 or M3 receptor reversed carbachol-induced increase in H_2_S production. Thus, although all M receptors are expressed in human bladder urothelium, only M1 and M3 appear to be involved in H_2_S production most likely for the specific anatomical localization. Indeed, in human urothelium, M1 receptor is localized on basal cells, M2 on umbrella cells, M3 and M4 homogenously and M5 with a decreasing gradient from luminal to basal cells^31^. The M1 receptor localization on basal cells that are anatomically strictly attached to detrusor muscle, suggests that H_2_S, once generated, can easily diffuse to the detrusor muscle contributing to its relaxation. Besides, our results suggest H_2_S as a possible candidate for UDRF. Indeed, previous studies demonstrated that M1 and M3 receptors stimulation induce the release of UDRF in rat bladder^23^. The identification of H_2_S as an UDRF in human bladder implies a role for this pathway in bladder tone and possibly in micturition. The urothelium impermeability is necessary for the normal bladder functioning, but at the same time represents limitations regarding the uptake of drugs. Therefore, the development of releasing-H_2_S drugs may represent a feasible therapeutic approach to bladder disorders. However our data strongly imply that the cholinergic stimulation triggers the detrusor muscle contraction but at the same time triggers the release of H_2_S by the urothelium. Therefore, it is feasible to hypothesize that in healthy condition a balance between these two signals e.g. contracting and relaxing effect contributes to bladder homeostasis. Conversely, in pathological condition, such as in overactive bladder, the detrusor contracting effect may predominate while in hyporeactive bladder the urothelium relaxing effect may prevail. To date, the knowledge of human bladder physiology is still unclear but our findings may open new frontiers in this field and may contextually offer the basis for novel pharmacological approaches in the management of bladder disorders.

## Methods

### Human tissue

Full thickness bladder dome samples were obtained from 15 patients, aged 61–73 years, affected with benign prostatic hyperplasia that underwent open prostatectomy. All patients presented urodynamic obstruction and large prostate volume (>80 ml). Patients were excluded if they presented bladder stones, urinary infections, detrusor areflexia and/or a history of urothelial cancer. The research has been carried out in accordance with the Declaration of Helsinki (2013) of the World Medical Association. The Ethical Committee of the Institution (School of Medicine and Surgery, University of Naples Federico II, via Pansini, 5; 80131, Naples, Italy) in which the work was performed has approved it. The subjects have given written informed consent to the work.

### Human bladder strips

The bladder sections (detrusor muscle *plus* urothelium), obtained from patients were excised from each donor sample, and longitudinal strips were isolated. Strips were suspended in organ chambers filled with Krebs buffer (37 °C, 95% oxygen and 5% carbon dioxide) with the following composition: sodium chloride,115.3 mM; potassium chloride, 4.9 mM; calcium chloride, 1.46 mM; magnesium sulfate, 1.2 mM; potassium dihydrogen phosphate, 1.2 mM; sodium bicarbonate, 25.0 mM; and glucose, 11.1 mM (Carlo Erba, Milan, Italy). The strips were connected to isometric force transducers (model 7002, Ugo Basile, Comerio, Italy) and a tension of 0.5 g was applied. The tension changes were registered and monitored by a computerized system (DataCapsule, Ugo Basile, Comerio, Italy). The tissues were equilibrated (60 min) and standardized by repeated contractions with carbachol (1 μM; Sigma, Milan, Italy). The strips with or without urothelium were challenged with carbachol (1 μM). The contraction was calculated as dyne/mg tissue and expressed as mean ± SE. The results were analyzed using Student’s t-test. In another setting of experiments, a concentration-response curve of carbachol (10 nM up to 30 μM) was performed in presence of urothelium and thereafter the curve was repeated in presence of vehicle, PAG, a CSE inhibitor (10 mM, Sigma, Milan, Italy), or AOAA, a CBS inhibitor (1 mM; Sigma, Milan, Italy) after 20 min of incubation. Data were calculated as delta percentage of increase in contraction between the two repeated concentration-response curve of carbachol. Data were expressed as mean ± SE and analyzed by using one way ANOVA. p < 0.05 was considered significant.

### Cell culture

The urothelial T24 cell line (American Type Culture Collection, ATCC, HTB-4) was cultured as previously described^32^.

### CBS silencing

To stably silence CBS, T24 cells were seeded the day before transfection in a 60 mm plate. Cells were transfected 24 hours later, with 2 μg of a CBS short hairpin (sh)RNA expressing vector purchased from Santa Cruz Biotechnology (Santa Cruz Biotechnology, Santa Cruz, CA, USA). CBS silenced cells (T24ΔCBS) were selected as previously described^33^.

The CBS depletion was evaluated by western blot using a rabbit polyclonal anti-CBS (Santa Cruz Biotechnology, DBA, Milan, Italy). The cell clone with lowest expression level was selected and used for further experiments. A short hairpin non-silencing construct was used as control.

### RT-PCR analyses

The mRNA level of M receptors (M1–M5) in urothelial tissue and in T24 cells was determined by using RT-semi-quantitative PCR (RT-PCR) method^34^. Total RNA was extracted from samples by using an ultrapure TRizol reagent (GibcoBRL) as directed by the manufacturer. RNA (2 μg) was reverse-transcribed to cDNA through SuperScript III First-Strand Synthesis system (iScript^TM^ cDNA Syntesis Kit, Biorad, Italy) according to the manufacturer’s protocol in the presence of random hexamers (5 μM), 20 U of RNasin (Promega), dNTP (1mM) for 1 h at 42 °C. RT-PCR was performed on 1 ml of the reverse transcription reaction mixture in a final volume of 50 ml with 2 U of Taq DNA Polymerase (VWR, Italy) and 1 mM of appropriate primers (Table 1). To obtain a linear amplification curves, the cDNA mixtures were subjected to denaturation at 95 °C for 1 min, annealing at 58 °C for 1 min for M2, M3 and β-actin and at 60 °C for M1, M4 and M5 and extension at 72 °C for 15 min.

### Human tissues and T24 cells treatments

In order to measure H_2_S production, human urothelial samples, T24 cells or T24ΔCBS were incubated with carbachol (Sigma-Aldrich, Milan, Italy) for 5 min at different concentrations (0.1, 1 and 10 μM). On the basis of these results, 1 μM was chosen as the optimal concentration. Thus, in another setting of experiments, human urothelial samples or T24 cells were pre-treated with AOAA (1 mM, for 20 min), a CBS inhibitor, and then challenged with carbachol (1 μM, for 5 min). The carbachol-induced effect was also evaluated in presence of telenzepine (10 nM, M1 antagonist), AFDX (100 nM, M2 antagonist), 4DAMP (100 nM, M3 antagonist) or PD102809 (100 nM, M4 antagonist). Finally, in order to investigate the underlying mechanism, T24 cells were pretreated with L-NIO, an endothelial nitric oxide synthase (eNOS) inhibitor (1 μM, Tocris UK) or KT5823, a PKG inhibitor (10 μM, Tocris UK) and thereafter stimulated with carbachol (1 μM, for 5 min).

### H_2_S determination

H_2_S production was evaluated as reported by d’Emmanuele di Villa Bianca and coworkers^35^. Human urothelial samples or T24 cells were homogenized with lysis buffer containing potassium phosphate buffer (100 mM, pH 7.4), sodium orthovanadate (10 mM) and proteases inhibitors. Homogenates were added for 40 min in a reaction mixture (total volume 500 μl) containing piridoxal-5′-phosphate (2 mM, 20 μl) and saline (20 μl) or L-cysteine (10 mM, 20 μl, Sigma-Aldrich, Milan, Italy) to measure enzyme activity i.e. H_2_S generation. The reaction was performed in parafilmed Eppendorf tubes and initiated by transferring tubes from ice to a water bath at 37 °C and carried out for 40 min. After incubation, zinc acetate (1%, 250 μl, Sigma-Aldrich) and trichloroacetic acid (10%, 250 μl) were added. Subsequently, N,N-dimethyl-p-phenylendiamine sulfate (DPD, 20 mM, Sigma-Aldrich) in HCl (7.2 M) and iron chloride (FeCl3, 30 mM, Sigma-Aldrich) in HCl (1.2 M) were added and optical absorbance of the solutions was measured after 20 min at a wavelength of 667 nm. All samples were assayed in duplicate and H_2_S concentration was calculated against a calibration curve of NaHS (3.9–250 μM, Sigma-Aldrich). Results were calculated as nmol/mg protein/min and expressed as mean ± SE. Data were analyzed by using one way ANOVA following by Bonferroni as post test. p < 0.05 was considered significant.

### cGMP measurement in T24 cells

In order to measure the cGMP content, T24 cells were treated with carbachol (0.1, 1 and10 μM) for 5 min and then immediately frozen. T24 cells were treated with 15 μl of 3.3 M HCl and then centrifuged (600×g for 10 min); the cGMP levels were measured in supernatants as previously reported^36^,^37^ by using an immune assay kit (Cayman; Vinci Biochem, Vinci, Italy). Results were calculated as pmol/ml and expressed as mean ± SE. Data were analyzed by using one way ANOVA following by Bonferroni as post test. p < 0.05 was considered significant.

### Western blot analysis in T24 cells

T24 cells treated with carbachol (0.1, 1 and 10 μM, for 5 min) were analyzed by western blotting in presence or in absence of KT5823 (10 μM), a PKG inhibitor (Tocris, UK). Protein samples (30 μg) were resolved by 12% SDS-PAGE and analyzed as previously described^38^,^39^.The membranes were incubated with rabbit polyclonal anti-CBS, (1:1000 Santa Cruz Biotechnology, Heidelberg, Germany), and anti-pCBS^Ser227^ (1:400, PRIMM srl, Milano, Italy)^21^. In another setting of experiments T24 cells or human urothelium were blotted for anti-eNOS (1:1000, BD Transduction, CA, USA). The target protein band intensity was normalized over the intensity of the housekeeping β-actin (1:5000, Sigma-Aldrich, Milan, Italy). Data were expressed as mean  ±  SE. Results were analyzed by ANOVA following by Bonferroni as post test. p  <  0.05 was considered significant.

## Additional Information

**How to cite this article**: d’Emmanuele di Villa Bianca, R.d.E.d.V. *et al*. Urothelium muscarinic activation phosphorylates CBS^Ser227^ via cGMP/PKG pathway causing human bladder relaxation through H_2_S production. *Sci. Rep.* **6**, 31491; doi: 10.1038/srep31491 (2016).

## Figures and Tables

**Table 1:** Primers used in PCR reactions.

Primers	Forward (5′-3′)	Reverse (5′-3′)
M Receptor 1 (M1)	GCTCTACTGGCGCATCTACC	TCCTTCTCCTGCTTCCGAGG
M Receptor 2 (M2)	CCAGTCAAGCGGACCACAAA	ATGCCGATAACGTCGGAAGA
M Receptor 3 (M3)	GGTCATACCGTCTGGCAAGT	GAAGGAGAATTCGGACCGGA
M Receptor 4 (M4)	GGCAGTTTGTGGTGGGTAAC	CGGGTGATTACTTCGTCTCG
M Receptor 5 (M5)	AGTTTCTCTCTGAGCCCACC	CCCAAGACTGAGACACTGGT
